# Changes in microstates of first-episode untreated nonsuicidal self-injury adolescents exposed to negative emotional stimuli and after receiving rTMS intervention

**DOI:** 10.3389/fpsyt.2023.1151114

**Published:** 2023-04-27

**Authors:** Lin Zhao, Dongdong Zhou, Jinhui Hu, Xiaoqing He, Xinyu Peng, Lingli Ma, Xinyi Liu, Wanqing Tao, Ran Chen, Zhenghao Jiang, Chenyu Zhang, Jing Liao, Jiaojiao Xiang, Qi Zeng, Linxi Dai, Qi Zhang, Su Hong, Wo Wang, Li Kuang

**Affiliations:** ^1^Department of Psychiatry, The First Affiliated Hospital of Chongqing Medical University, Chongqing, China; ^2^Mental Health Center, University-Town Hospital of Chongqing Medical University, Chongqing, China

**Keywords:** nonsuicidal self-injury, adolescents, repetitive transcranial magnetic stimulation, EEG microstates, emotional stimulation tasks

## Abstract

**Background:**

Nonsuicidal self-injury (NSSI) is a common mental health threat in adolescents, peaking in adolescence with a lifetime prevalence of ~17%–60%, making it a high-risk risk factor for suicide. In this study, we compared changes in microstate parameters in depressed adolescents with NSSI, depressed adolescents, and healthy adolescents during exposure to negative emotional stimuli, and further explored the improvement of clinical symptoms and the effect of microstate parameters of repetitive transcranial magnetic stimulation (rTMS) in depressed adolescents with NSSI, and more evidence was provided for potential mechanisms and treatment optimization for the occurrence of NSSI behaviors in adolescents.

**Methods:**

Sixty-six patients with major depressive disorder (MDD) exhibiting NSSI behavior (MDD + NSSI group), 52 patients with MDD (MDD group), and 20 healthy subjects (HC group) were recruited to perform neutral and negative emotional stimulation task. The age range of all subjects was 12–17 years. All participants completed the Hamilton Depression Scale, the Patient Health Questionnaire-9, the Ottawa Self-Injury Scale and a self-administered questionnaire to collect demographic information. We provided two different treatments to 66 MDD adolescents with NSSI; 31 patients received medication and completed post-treatment scale assessments and EEG acquisitions, and 21 patients received medication combined with rTMS and completed post-treatment scale assessments and EEG acquisitions. Multichannel EEG was recorded continuously from 64 scalp electrodes using the Curry 8 system. EEG signal preprocessing and analysis was performed offline, using the EEGLAB toolbox in MATLAB. Use the Microstate Analysis Toolbox in EEGLAB for segmentation and computation of microstates, and calculate a topographic map of the microstate segmentation of the EEG signal for a single subject in each dataset, and four parameters were obtained for each microstate classification: global explained variance (GEV), mean duration (Duration), average number of occurrences per second (Occurrence), and average percentage of total analysis time occupied (Coverage), which were then statistically analyzed.

**Results:**

Our results indicate that MDD adolescents with NSSI exhibit abnormalities in MS 3, MS 4, and MS 6 parameters when exposed to negative emotional stimuli compared to MDD adolescents and healthy adolescents. The results also showed that medication combined with rTMS treatment improved depressive symptoms and NSSI performance more significantly in MDD adolescents with NSSI compared to medication treatment, and affected MS 1, MS 2, and MS 4 parameters in MDD adolescents with NSSI, providing microstate evidence for the moderating effect of rTMS.

**Conclusion:**

MDD adolescents with NSSI showed abnormal changes in several microstate parameters when receiving negative emotional stimuli, and compared to those not receiving rTMS treatment, MDD adolescents with NSSI treated with rTMS showed more significant improvements in depressive symptoms and NSSI performance, as well as improvements in EEG microstate abnormalities.

## Introduction

Nonsuicidal self-injury (NSSI) is a common mental health problem in adolescents that peaks during adolescence (1). Studies have found that NSSI has a high lifetime prevalence ranging from ~17%–60% and a multifactorial etiology, including social factors, interpersonal stress, neurobiological background, emotional dysregulation, and traumatic childhood experiences (2). Notably, NSSI behaviors are associated with a large number of negative states, including high levels of negative emotions, interpersonal tension, and academic stress (3). NSSI behaviors, although not usually fatal, have been shown in both large cross-sectional and longitudinal studies to be at significantly increased risk for suicidal ideation and suicide attempts, particularly in recurrent NSSI (4–6). Repeated self-injury implies that adolescents with NSSI lack control over inappropriate or unwanted behavior, and this ability to control inappropriate or unwanted behaviors is inhibitory control. Previous research has demonstrated that inhibitory control is impaired in the context of negative emotions (7). Thus, exposure to negative emotional stimuli may lead to the occurrence of impulsive behaviors in adolescents, which include NSSI behaviors (8, 9). Therefore, a better understanding of NSSI in adolescents, early identification for prevention as well as timely intervention is crucial for current and future prediction of suicide risk (10).

However, there is a lack of research on the treatment of adolescents with NSSI, or those with other psychiatric disorders that frequently co-occur with NSSI (11). One study found the odds of co-existing depression in adolescents with NSSI of ~41.6% (12). In general, treatment for NSSI should always include interventions for other comorbid psychiatric disorders, if present. Much of the previous research has focused on psychotherapy and psychopharmacological treatment. However, because of the small number of published studies, no specific treatment has been established as superior or the treatment of choice at this stage (13). Because of the long duration of psychotherapy and the potential adverse effects of medication, treatment adherence is low among adolescents with NSSI. Repetitive transcranial magnetic stimulation (rTMS), a non-invasive neuromodulation technique, has shown good clinical efficacy in depression (14). A longitudinal study of rTMS for depressive disorders in adolescents and adults found higher rates of symptom improvement and remission in adolescent patients than in adults and no safety or tolerability issues (15). However, existing studies of rTMS for depression have focused more broadly on its effects on overall clinical outcomes, including changes in mood symptoms, rather than on its effects on impulsivity and self-injury (16). Notably, a recent consensus statement by a European expert group on rTMS did not even include self-injury as a short-term or long-term goal (17). Therefore, there is a need and an opportunity to correct this gap and translate cognitive neuroscience activities into treatment options. In a recent study involving 377 in patients treated with a 3-day intensive 10 Hz rTMS in the left dorsolateral prefrontal cortex (DLPFC), patients showed rapid improvement on the Beck Scale of Suicidal Ideation (BSSI) (18). Thus, it is reasonable to speculate that rTMS may be an effective treatment for nonsuicidal self-injury in adolescent depression.

Multichannel EEG is a powerful tool for exploring the spatiotemporal activity of the human brain and has been applied to study neural activity in the brain because of its advantage of displaying neurodynamic at high temporal resolution. Unlike traditional ERP analysis, microstate (MS) analysis (19) allows exploration and comparison of the activation of brain activity by precisely quantifying temporal features such as onset time or duration. Microstate analysis, a technique first proposed by Lehmann (20), utilizes the high temporal resolution of the EEG to segment the EEG signal into short continuous time segments characterized by a sub-steady state scalp topology corresponding to a consistent synchronous activation period of a large-scale neuronal network (21). Lehmann et al. propose the concept of microstates, as “thought atoms,” which suggests that they are basic components of information processing, whether generated spontaneously generated or in response to a stimulus (20). This view is consistent with the suggestion that neurocognitive networks evolve through a series of coordinated quasi-stable states rather than continuous neuronal activity (22). For stimulus-induced task microstates, each microstate represents a specific information processing step from perception to action (23). Based on this view, different methods have been used to objectively and automatically define different microstates using modules of the EEGLAB’s Microstate Analysis Toolkit such as “Microstate Segmentation” and “Map Fitting” (24). These modules can also be used to statistically assess the specificity of certain microstates under given experimental conditions. Microstates can be measured quantitatively using metrics such as global explained variance, mean duration, frequency of occurrence per unit time, and temporal coverage. We anticipate that microstate analysis may provide new perspectives or evidence for identifying biomarkers in adolescents with NSSI or to understand the potential mechanisms of rTMS in treating the adolescent population with NSSI.

Therefore, this study aimed to address the following questions: (1) how do the microstate characteristics of adolescents with NSSI change after exposure to negative emotional stimuli? and (2) how does rTMS treatment affect the microstate characteristics of adolescents with NSSI? Addressing these questions will provide new insights into the neural basis of NSSI behavior in adolescence and potential markers for development of effective treatment modalities.

## Methods

### Participants

This study included 20 healthy subjects (HC group, 8 males, 12 females, mean age: 15.45 years), 52 adolescents with depression (MDD group, also as a patient control group, 20 males, 32 females, mean age: 15.31 years), and 66 MDD adolescents with NSSI (MDD + NSSI group, 12 males, 54 females, mean age: 14.33 years). These patients were recruited from the outpatient and inpatient wards of the First Affiliated Hospital of Chongqing Medical University and the University City Hospital of Chongqing Medical University. All patients were diagnosed with major depressive disorder according to the ICD-10 diagnostic criteria. NSSI was determined based on the Diagnostic and Statistical Manual of Mental Disorders, Fifth Edition (DSM-5) (25) diagnostic criteria for nonsuicidal self-injury and the Ottawa Self-Injury Scale (26). Healthy subjects were recruited from secondary schools and matched as closely as possible to the patient group in terms of sex and age. All participants were fully informed of the procedures and purpose of the study and provided written consent before the start of the study. All procedures met ethical standards and were approved by the Ethics Committee of Chongqing Medical University. All subjects were right-handed, with normal or corrected vision and normal hearing. Exclusion criteria included history of neurological or other psychiatric disorders other than depression, history of chronic substance use, learning disabilities, and head injury resulting in loss of consciousness. Before conducting the experiment, all subjects were interviewed using the MINI-International Neuropsychiatric Interview (M.I.N.I. KID 5.0) (27), followed by a scale assessment by two trained psychiatrists.

### Questionnaires

All participants completed the Hamilton Depression Scale (HAMD-17) (28) and the Patient Health Questionnaire-9 (PHQ-9) (29) to determine participants’ levels of depressive symptoms, and the Ottawa Self-Injury Scale to determine details of the severity of self-injury behaviors. In addition, all participants completed a self-administered questionnaire to collect demographic information.

### Negative emotional stimuli task

#### Stimuli

The stimuli selected in this study were emotional face pictures, including one neutral emotional picture and eight different negative emotional pictures. The different negative emotional pictures were used to avoid the confounding repetitive effects of the stimuli. Both the neutral and negative emotional pictures were selected from the Chinese Facial Affective Picture System (CFAPS) (30), which controls the arousal, luminance, color, and other relevant attributes of the emotional pictures using standardized set of stimuli. All images were identical in size and resolution.

#### Procedure

**Figure 1:**
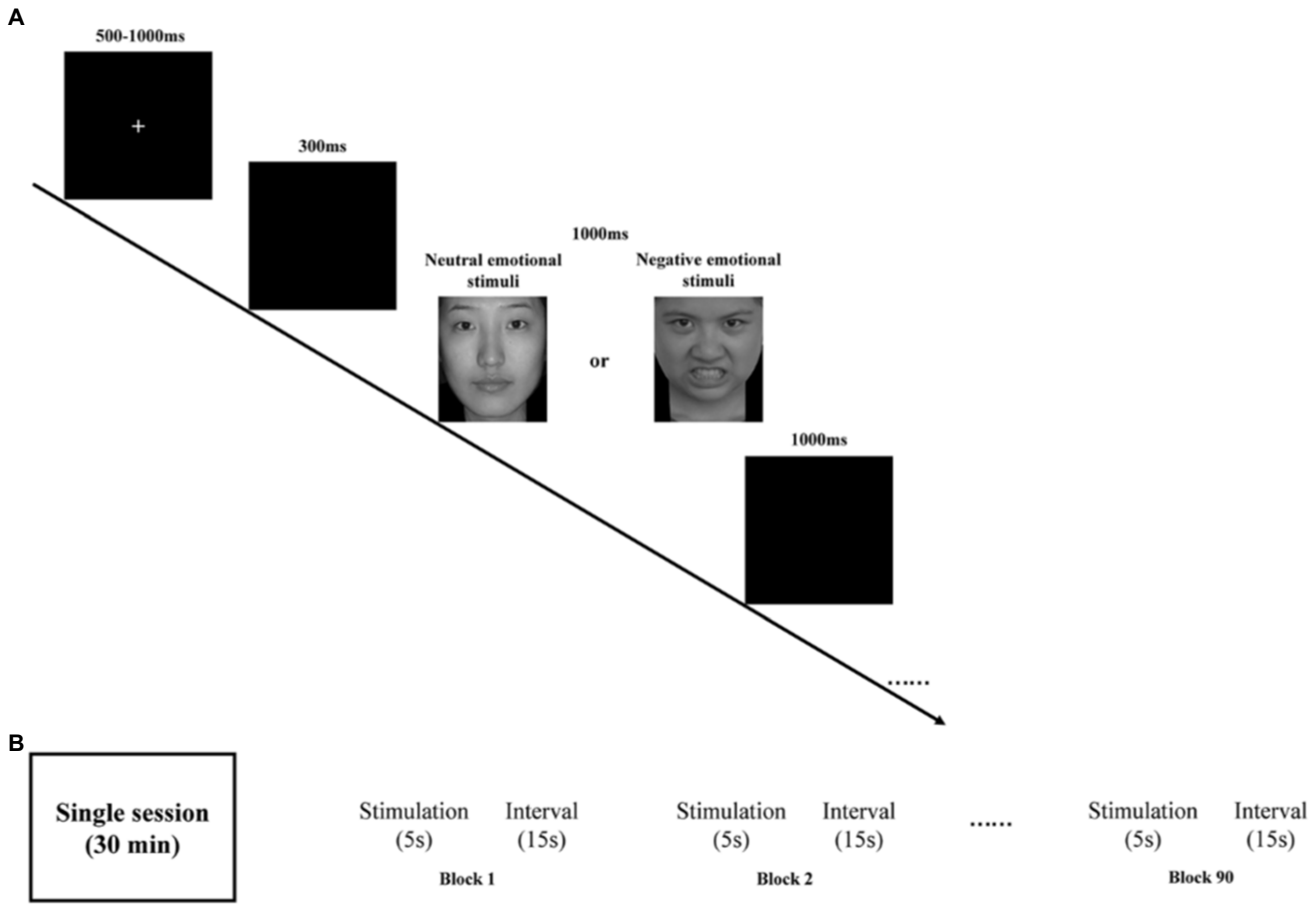
**(A)** Schematic illustration of the experimental procedure and examples of stimuli. **(B)** rTMS program: each session is 30 min and consists of 90 blocks. Each block has 5 s stimulation and 15 s stimulus intervals.

Participants, seated at ~60 cm from a computer screen, were presented with a stimulus task with E-Prime 3.0. Both neutral and negative emotional stimuli task were used in our study. The task is shown in Figure 1A.

Briefly, all emotional pictures were presented on a black background. By randomly disrupting the presentation of the stimuli, all emotional pictures appeared a total of 200 times, including 150 times for neutral emotional picture 50 times for negative emotional picture. To control the effect of onset, at the beginning of each stimulus presentation, a fixed intersection of randomly selected durations of 500–1000 ms was initially presented on the computer screen, followed by a blank screen for 300 ms. Subsequently, a randomly presented emotion pictures were randomly presented for a duration of 1000 ms before disappearing or ending early based on the participant’s response. Finally, a blank screen was presented again for 1000 ms on the computer screen. Participants were asked to press either “1” button when presented with a neutral emotion picture or the “2” button when presented with a negative emotion as quickly and accurately as possible. Participants were required to achieve at least 80% accuracy in the exercise before the start of the formal trial.

#### EEG acquisition, preprocessing, and EEG segmentation

Multichannel EEG was recorded continuously from 64 scalp electrodes using the Curry 8 system. For accurate measurement, these electrodes were placed according to the international 10/20 system. One vertical EEG electrode was placed above and below the left eye and one horizontal EEG electrode was placed at the orbital canthus of the left and right eyes to allow for monitoring of eye movements and subsequent removal of eye movement artifacts from the recordings. All channels were digitally sampled at 1000 Hz and the reference electrode between Cz and Cpz was chosen as an online reference. Bandpass filtering was set to 0.5–80 Hz. Data acquisition did not start until all impedance values were below 5 kΩ.

**Figure 2:**
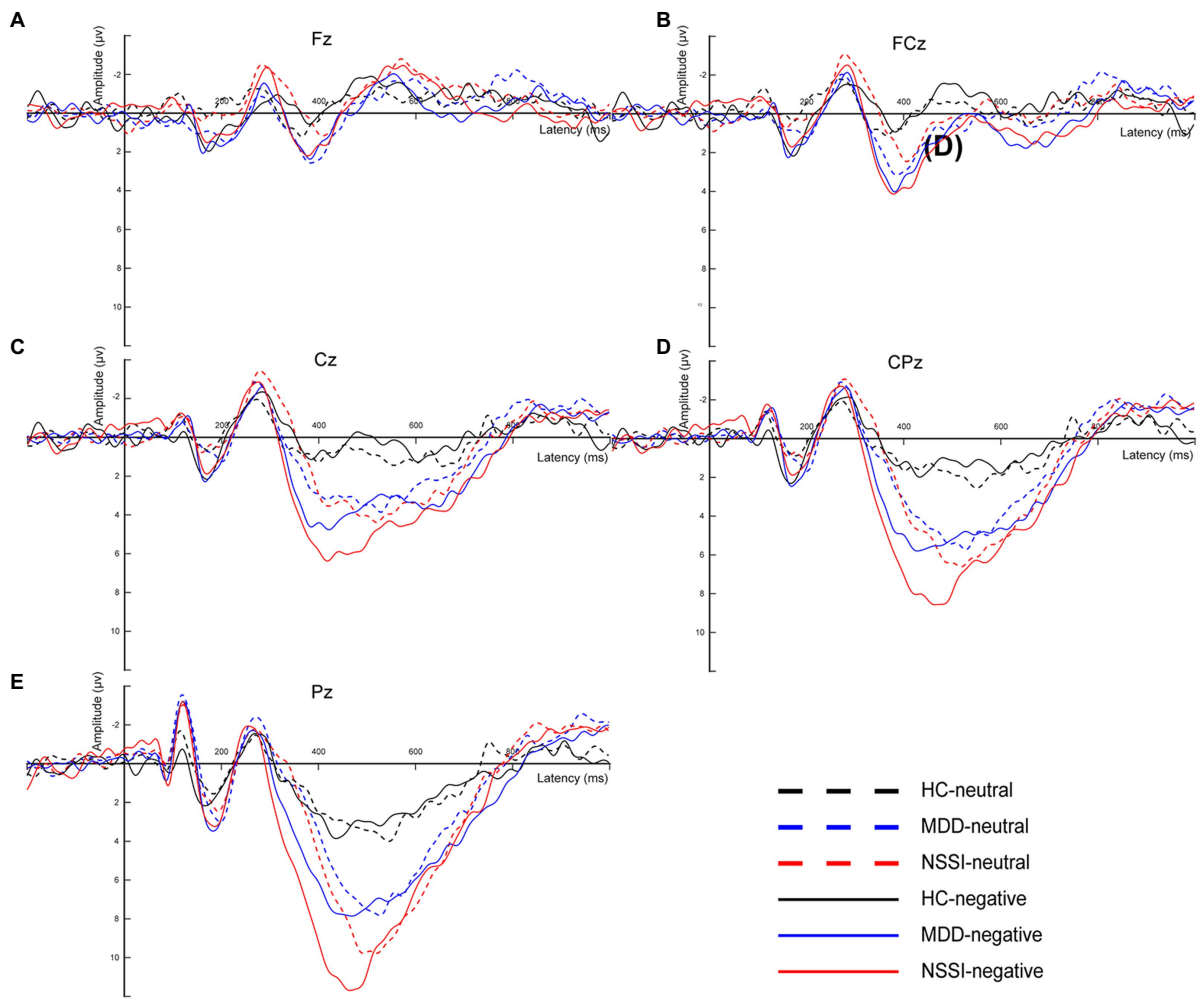
**(A)** The average difference waveforms at Fz electrode under neutral and negative emotional cues in the HC, MDD and MDD+NSSI groups. **(B)** The average difference waveforms at FCz electrode under neutral and negative emotional cues in the HC, MDD and MDD+NSSI groups. **(C)** The average difference waveforms at Cz electrode under neutral and negative emotional cues in the HC, MDD and MDD+NSSI groups. **(D)** The average difference waveforms at CPz electrode under neutral and negative emotional cues in the HC, MDD and MDD+NSSI groups. **(E)** The average difference waveforms at Pz electrode under neutral and negative emotional cues in the HC, MDD and MDD+NSSI groups. HC, healthy control; MDD, major depressive disorder; MDD+NSSI, MDD with nonsuicidal self-injury.

EEG signals were preprocessed and analyzed offline using the EEGLAB toolbox in MATLAB (31). First, to obtain cleaner and accurate data, we resampled all EEG data with the sampling rate set to 500 Hz and performed secondary filtering with the frequency range set to 0.1–30 Hz. Useless electrodes such as EKG, EMG, CB1, and CB2 were also removed. The data were reviewed to remove incorrect responses as well as non-responsive time segments, retaining only those with correct responses. Subsequently, the EEG data were re-segmented to generate segments, each comprising 200 ms before and 1000 ms after stimulation. Segments with large artifacts were rejected and poor channels were interpolated. Independent component analysis (ICA) was used to remove artifact components, mainly including blinks, horizontal eye movements, and muscle artifacts (32). To identify individual responses to emotional stimuli, EEG data segments were selected for all correct responses to negative emotional stimuli and the EEG data for this condition were averaged to obtain the mean EEG signal for each subject. The average difference waveforms on the midline electrodes were also plotted for comparison with the EEG microstate classification (Figure 2).

#### EEG microstate segmentation and computation

**Figure 3:**
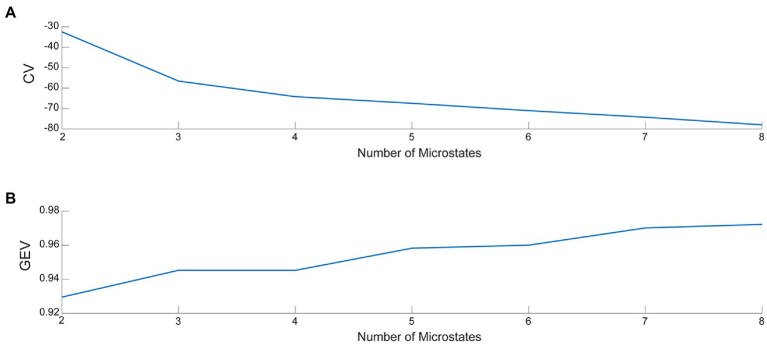
**(A)** The value of CV for various microstate classifications. **(B)** The value of GEV for various microstate classifications.

Microstate analysis was performed using the Microstate analysis toolbox (33) in EEGLAB. First, we used the toolbox to load the pre-processed EEG data into EEGLAB and examined the data structure bodies. The average EEG signal was obtained by averaging all channel EEG data for each subject across periods and then normalizing the data. Subsequently, the mean EEG signal was segmented for microstates. A prototype topographic map of the EEG signal was determined using the k-means clustering algorithm. The k-means clustering analysis is a classical pattern recognition method, involving an iterative process that starts with an initial guess of the map and terminates when the differences are negligible in successive iterations (34). Because of differences in the number of iterations, the results of k-means clustering analysis may differ slightly from one run to another. It is not recommended to set the number of iterations too low. Therefore, the number of iterations of k-means in this study was set to 1000. The optimal number of topographic maps calculated, i.e., the microstate classification, was also determined using the cross-validation method (CV) and the global explained variance (GEV). The larger the GEV and the smaller the CV, the better the microstate classification (33). The number of microstate classifications was plotted (Figure 3). The determined microstate classification topographies were fitted to each subject’s EEG signal to calculate a topographic map of the microstate segmentation of the EEG signal for a single subject in each dataset. In addition, global explained variance (“GEV”), mean duration (“Duration”), average number of occurrences per second (“Occurrence”), and average percentage of total analysis time occupied (“Coverage”) for the four microstate parameters were determined as follows:

(1) GEV: the sum of the explained variance, weighted by the global field power at each moment.
(2) Duration: the average time a single microstate was classified as present.
(3) Coverage: the percentage of time covered by a single microstate classification.
(4) Occurrence: the number of occurrences of a single microstate classification per unit time.

### Treatment

We treated 66 MDD adolescents with NSSI for 8 weeks. Among them, 41 patients received medication only and 25 patients received medication combined with rTMS. Scale assessments and EEG data collection were repeated for these participants at the end of treatment. However, 14 treated adolescents were unable to complete the post-treatment scale assessment and EEG collection and were therefore excluded from the data set. Ultimately, 31 patients completed medication follow-up and 21 patients completed medication combined with rTMS treatment follow-up.

The medication of choice was selective serotonin reuptake inhibitors (SSRIs), such as sertraline, at a therapeutic dose of 150 mg/day. The widely recognized figure-of-eight coil was used for rTMS treatment targeting the left dorsolateral prefrontal cortex region. The following simulation parameters (Figure 1B) were used: location (left DLPFC, EEG International 10–20 system, F3 electrode), intensity (100% of individual resting motor threshold), stimulation frequency (10 Hz), 4500 stimulations per session (90 blocks, 50 trains), single train duration (5 s), inter-train interval (15 s), and stimulation period (once per day, 5 days per week for 4 weeks). These parameters are consistent with published safety guidelines for rTMS (35, 36).

### Data analysis

Statistical analyses were conducted using the Statistical Package for the Social Sciences (SPSS) version 25.0. We first conducted cross-sectional analyses using ANOVA or Chi-square tests for demographic data of participants in each group, one-way ANOVA for scale data of participants in each group, and 2 × 3 repeated-measures ANOVA for microstate parameters, where emotional cues were used as within-group factors (two levels: neutral emotional cues, negative emotional cues) and group as between-group factors (three levels: HC group, MDD group, MDD + NSSI group). Then, we performed a 2 × 2 repeated-measures ANOVA on the scale data of participants in the MDD + NSSI group who received medication treatment or medication combined with rTMS treatment where treatment time was used as a within-group factor (two levels: before treatment, after treatment) and treatment method as a between-group factor (two levels: medication treatment, medication combined with rTMS treatment); a 2 × 2 × 2 repeated-measures ANOVA was performed on microstate parameters, where emotional cues were used as within-group factor 1 (two levels: neutral emotional cues, negative emotional cues), treatment time was used as a within-group factor 2 (two levels: before treatment, after treatment), and treatment method as a between-group factor (two levels: medication treatment, medication combined with rTMS treatment). All significant interaction effects were further analyzed using simple effects analysis. Post-hoc comparison was performed for significant main or between-group effects using the Bonferroni–Holm method. In cases where sphericity could not be assumed, statistical values were reported using the Greenhouse–Geisser correction. The alpha level of significance was set at *p* < 0.05.

## Results

### Cross-sectional analysis

#### Demographic and baseline clinical characteristic

The demographic and clinical characteristics of the three groups at the baseline are shown in Table 1. Age and sex were significantly different in the three groups (*p* = 0.002, *p* = 0.025). Subsequently, to control for the effects of age and sex, age and sex were used as covariates in the follow-up microstate analysis. There were significant group differences in the HAMD and PHQ-9 scores (all *p* < 0.001). Compared with HCs, participants with MDD and MDD + NSSI had significantly higher levels of depression (all *p* < 0.001). The NSSI characteristics, including NSSI numbers, NSSI first onset age and NSSI types, of the participants in the MDD + NSSI group are shown in Table 1.

**Table 1:** Demographic and clinical characteristics of participants in the three groups at the baseline.

	HC group (*n* = 20) *M* ± SD	MDD group (*n* = 52) *M* ± SD	MDD + NSSI group (*n* = 66) *M* ± SD	*F/χ* ^2^	*p* value
Age (years)	15.45 ± 2.282	15.31 ± 1.449	14.33 ± 1.601	6.464	**0.002**
Sex (male/female)	8/12	20/32	12/54	7.190	**0.025**
HAMD scores	1.30 ± 1.809	22.62 ± 3.448	23.14 ± 4.220	295.378	**<0.001**
PHQ-9 scores	1.50 ± 2.115	19.60 ± 3.610	20.14 ± 3.586	246.842	**<0.001**
NSSI number	/	/	10.26 ± 3.763		
NSSI first onset age	/	/	12.77 ± 1.644		
NSSI type					
Cutting			66/66		
Pinching			13/66		
Biting			4/66		
Knocking			10/66		
Burning			1/66		

HC, healthy controls; MDD, major depressive disorder; MDD + NSSI, MDD with nonsuicidal self-injury; HAMD, Hamilton Depression Scale; PHQ-9, Patient Healthcare Questionnaire.

#### Microstate parameters

**Figure 4:**
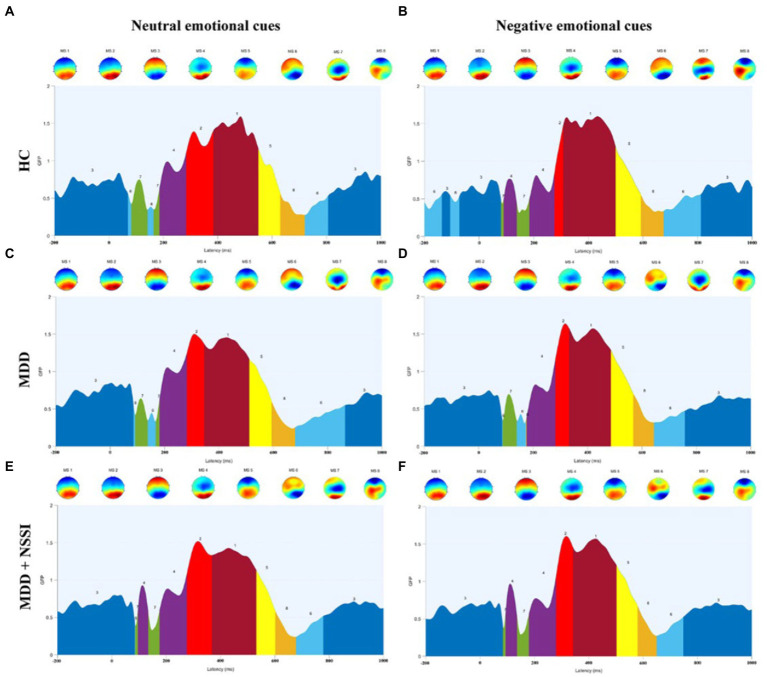
**(A)** Microstate categories of the HC group under neutral emotional cues. **(B)** Microstate categories of the HC group under negative emotional cues. **(C)** Microstate categories of the MDD group under neutral emotional cues. **(D)** Microstate categories of the MDD group under negative emotional cues. **(E)** Microstate categories of the MDD + NSSI group under neutral emotional cues. **(F)** Microstate categories of the MDD + NSSI group under negative emotional cues. HC, healthy control; MDD, major depressive disorder; MDD + NSSI, MDD with nonsuicidal self-injury.

When age and gender were included as covariates, the ANOVA results (see Table 2; Figure 4) showed that GEVs of all three groups were above 79%, which was highly explainable. There was a significant between-group main effect (*F*(2, 133) = 6.742, *p* = 0.002, *η*_p_^2^ = 0.092) for MS 3 duration, a significant cue × group interaction effect (*F*(2, 133) = 5.246, *p =* 0.006, *η*_p_^2^ = 0.073) and a significant between-group main effect (*F*(2, 133) = 9.746, *p* < 0.001, *η*_p_^2^ = 0.128) for MS 3 coverage; and there was a significant cue × group interaction effect (*F*(2, 133) = 3.369, *p =* 0.037, *η*_p_^2^ = 0.048) and a borderline significant between-group main effect (*F*(2, 133) = 2.935, *p =* 0.057, *η*_p_^2^ = 0.042) for MS 4 coverage, and a significant cue × group interaction effect (*F*(2, 133) = 5.669, *p =* 0.004, *η*_p_^2^ = 0.079) and a significant between-group main effect (*F*(2, 133) = 6.640, *p =* 0.002, *η*_p_^2^ = 0.091) for MS 4 occurrence; and there was a significant between-group main effect (*F*(2, 133) = 5.263, *p =* 0.006, *η*_p_^2^ = 0.073) for MS 6 duration, a significant cue × group interaction effect (*F*(2, 133) = 4.547, *p =* 0.012, *η*_p_^2^ = 0.064) and a significant between-group main effect (*F*(2, 133) = 10.191, *p* < 0.001, *η*_p_^2^ = 0.133) for MS 6 coverage, and a borderline significant between-group main effect (*F*(2, 133) = 2.901, *p =* 0.058, *η*_p_^2^ = 0.042) for MS 6 occurrence. No cue main effect, between-group main effect, or cue × group interaction effect was found for any of the other microstates.

**Table 2:** Microstate parameters of HC group, MDD group, and MDD + NSSI group exposed to neutral emotional cues and negative emotional cues.

Microstate parameters			HC group (*n* = 20) *M* ± SD	MDD group (*n* = 52) *M* ± SD	MDD + NSSI group (*n* = 66) *M* ± SD	Cue main effect *F*(*p*)	Between-group main effect *F*(*p*)	Cue × Group interaction effect *F*(*p*)
GEV		Neutral	0.824 ± 0.071	0.802 ± 0.121	0.819 ± 0.068	1.028 (0.312)	0.798 (0.452)	0.135 (0.874)
		Negative	0.821 ± 0.073	0.793 ± 0.123	0.814 ± 0.068			
Duration	MS3	Neutral	154.558 ± 63.305	191.082 ± 88.582	205.392 ± 76.819	0.126 (0.723)	6.742 (**0.002**)	0.596 (0.553)
		Negative	152.192 ± 75.408	212.864 ± 80.701	224.472 ± 64.911			
	MS6	Neutral	102.350 ± 73.351	96.531 ± 74.048	75.273 ± 49.292	0.015 (0.903)	5.263 (**0.006**)	1.494 (0.228)
		Negative	136.625 ± 82.443	95.571 ± 67.874	85.697 ± 64.675			
Coverage	MS3	Neutral	0.346 ± 0.109	0.372 ± 0.125	0.413 ± 0.093	0.132 (0.717)	9.746 (**<0.001**)	5.246 (**0.006**)
		Negative	0.307 ± 0.124	0.426 ± 0.094	0.448 ± 0.094			
	MS4	Neutral	0.082 ± 0.073	0.091 ± 0.051	0.107 ± 0.055	2.404 (0.123)	2.935 (**0.057**)	3.369 (**0.037**)
		Negative	0.081 ± 0.051	0.064 ± 0.050	0.098 ± 0.055			
	MS6	Neutral	0.145 ± 0.107	0.144 ± 0.114	0.103 ± 0.076	0.090 (0.765)	10.191 (**<0.001**)	4.547 (**0.012**)
		Negative	0.202 ± 0.122	0.122 ± 0.081	0.091 ± 0.070			
Occurrence	MS4	Neutral	0.875 ± 0.504	1.058 ± 0.574	1.212 ± 0.550	0.126 (0.723)	6.640 (**0.002**)	5.669 (**0.004**)
		Negative	1.125 ± 0.677	0.769 ± 0.518	1.174 ± 0.506			
	MS6	Neutral	1.417 ± 0.979	1.314 ± 0.953	1.212 ± 0.803	0.001 (0.973)	2.901 (**0.058**)	1.298 (0.277)
		Negative	1.542 ± 0.867	1.330 ± 0.966	0.960 ± 0.571			

The results of further simple effects analysis showed that the MS 3 duration and coverage in the MDD + NSSI group under negative emotional cues were significantly greater than those in the HC group (224.472 ± 64.911 vs. 152.192 ± 75.408, 0.448 ± 0.094 vs. 0.307 ± 0.124, all *p* < 0.05), and greater than in the MDD group, but the latter difference was not significant. And the MS 3 duration and coverage under negative emotional cues in the MDD + NSSI group were significantly greater than those under neutral emotional cues (224.472 ± 64.911 vs. 205.392 ± 76.819, 0.448 ± 0.094 vs. 0.413 ± 0.093, all *p* < 0.05). The MS 4 coverage and occurrence in the MDD + NSSI group under negative emotional cues were significantly greater than those in the MDD group (0.098 ± 0.055 vs. 0.064 ± 0.050, *p* = 0.002; 1.174 ± 0.506 vs. 0.769 ± 0.518, *p* < 0.001) and greater than those in the HC group, but the differences compared with the HC group were not statistically significant. In addition, the MS 6 duration, coverage, and occurrence in the MDD + NSSI group under negative emotional cues were significantly lower than those in the HC group (85.697 ± 64.675 vs. 136.625 ± 82.443, *p* = 0.005; 0.091 ± 0.070 vs. 0.202 ± 0.122, *p* < 0.001; 0.960 ± 0.571 vs. 1.542 ± 0.867, *p* = 0.007), and the MS 6 coverage and occurrence in the MDD + NSSI group were significantly lower than those in the MDD group (0.091 ± 0.070 vs. 0.122 ± 0.081, *p* = 0.032; 0.960 ± 0.571 vs. 1.330 ± 0.966, *p* = 0.022), and the MS 6 occurrence under negative emotional cues in the MDD + NSSI group was significantly lower than those under neutral emotional cues (0.960 ± 0.571 vs. 1.212 ± 0.803, *p* = 0.038).

### Longitudinal analysis

#### Changes in HAMD scores, PHQ-9 scores, and NSSI scores after receiving different interventions in the MDD + NSSI group

A repeated-measures ANOVA (see Table 3) on depression scores and NSSI scores in the MDD + NSSI group, using treatment method as a between-group factor and treatment time as a within-group factor, showed that there was a significant time × group effect for HAMD scores [*F*(1, 50) = 5.416, *p =* 0.024, *η*_p_^2^ = 0.098], PHQ-9 scores [*F*(1, 50) = 6.482, *p =* 0.014, *η*_p_^2^ = 0.115], and NSSI scores [*F*(1, 50) = 11.882, *p =* 0.001, *η*_p_^2^ = 0.192]; and a significant time main effect for HAMD scores [*F*(1, 50) = 223.063, *p* < 0.001, *η*_p_^2^ = 0.817], PHQ-9 scores [*F*(1, 50) = 178.727, *p* < 0.001, *η*_p_^2^ = 0.781], and NSSI scores [*F*(1, 50) = 225.491, *p* < 0.001, *η*_p_^2^ = 0.819]; and a significant between-group main effect for HAMD scores [*F*(1, 50) = 8.560, *p* = 0.005, *η*_p_^2^ = 0.146], PHQ-9 scores [*F*(1, 50) = 6.730, *p* = 0.012, *η*_p_^2^ = 0.119], and NSSI scores [*F*(1, 50) = 7.754, *p* = 0.008, *η*_p_^2^ = 0.134]. Further simple effects analysis revealed that participants who received medication combined with rTMS exhibited lower HAMD scores (8.381 ± 5.084 vs. 12.936 ± 3.829, *p* = 0.001), PHQ-9 scores (7.667 ± 5.713 vs. 11.968 ± 4.167, *p* = 0.001), and NSSI scores (0.238 ± 0.53 vs. 0.871 ± 0.718, *p* < 0.001) compared with those who received medication treatment.

**Table 3:** Changes in depression scores and NSSI scores before and after treatment in the MDD + NSSI group after receiving different treatment.

Clinical scales		Medication treatment (*n* = 31) *M* ± SD	Medication + rTMS treatment (*n* = 21) *M* ± SD	Time main effect *F*(*p*)	Between-group main effect *F*(*p*)	Time × Group interaction effect *F*(*p*)
HAMD	Before	23.612 ± 4.248	23.000 ± 4.336	223.063 (**<0.001**)	8.560 (**0.005**)	5.416 (**0.024**)
	After	12.936 ± 3.829	8.381 ± 5.084			
PHQ-9	Before	20.290 ± 3.175	19.905 ± 3.780	178.727 (**<0.001**)	6.730 (**0.012**)	6.482 (**0.014**)
	After	11.968 ± 4.167	7.667 ± 5.713			
NSSI	Before	2.065 ± 0.250	2.143 ± 0.359	225.491 (**<0.001**)	7.754 (**0.008**)	11.882 (**0.001**)
	After	0.871 ± 0.718	0.238 ± 0.539			

#### Changes in microstate parameters after receiving different interventions in the MDD + NSSI group

**Figure 5:**
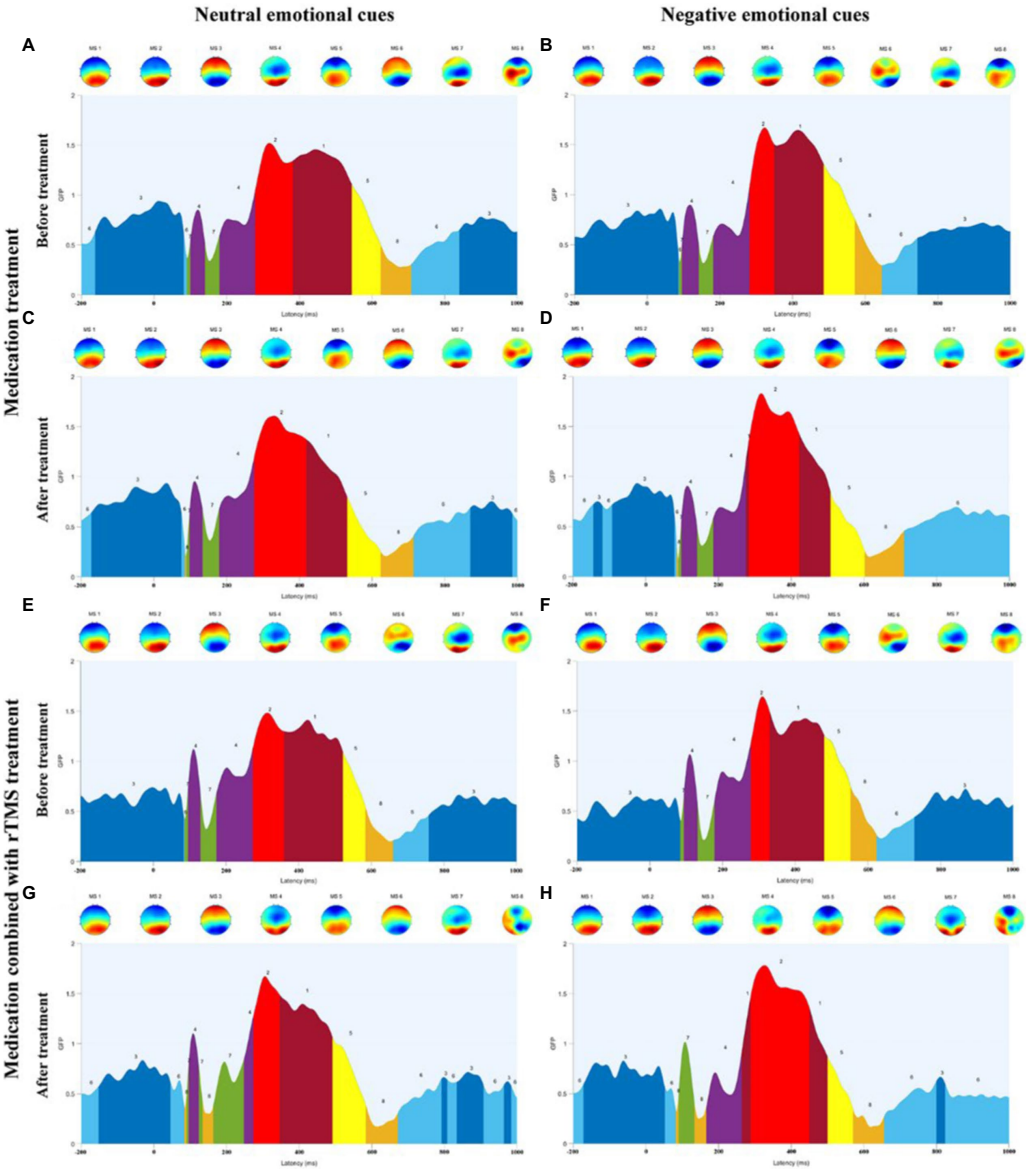
**(A)** Microstate categories of MDD adolescents with NSSI under neutral emotional cues before medication treatment. **(B)** Microstate categories of MDD adolescents with NSSI under negative emotional cues before medication treatment. **(C)** Microstate categories of MDD adolescents with NSSI under neutral emotional cues after medication treatment. **(D)** Microstate categories of MDD adolescents with NSSI under negative emotional cues after medication treatment. **(E)** Microstate categories of MDD adolescents with NSSI under neutral emotional cues before medication combined with rTMS treatment. **(F)** Microstate categories of MDD adolescents with NSSI under negative emotional cues before medication combined with rTMS treatment. **(G)** Microstate categories of MDD adolescents with NSSI under neutral emotional cues after medication combined with rTMS treatment. **(H)** Microstate categories of MDD adolescents with NSSI under negative emotional cues after medication combined with rTMS treatment.

A repeated-measures ANOVA (see Table 4; Figure 5) was performed on microstate parameters in the MDD + NSSI group using treatment method as a between-group factor, treatment time as a within-group factor1, and emotional cues as a within-group factor2, showed that GEVs in the MDD + NSSI group receiving the two different treatment methods was above 80% for both neutral and negative emotional cues before and after treatment, which was highly explainable. There was a significant cue × group interaction effect (*F*(1, 50) = 4.334, *p =* 0.042, *η*_p_^2^ = 0.080) for MS 1 duration, a significant cue × group interaction effect (*F*(1, 50) = 6.883, *p =* 0.012, *η*_p_^2^ = 0.121) and a significant time × cue × group interaction effect (*F*(1, 50) = 4.778, *p =* 0.034, *η*_p_^2^ = 0.087) for MS 2 duration, and a significant cue × group interaction effect (*F*(1, 50) = 12.737, *p =* 0.001, *η*_p_^2^ = 0.203) for MS 2 coverage; there was a significant time main effect (*F*(1, 50) = 46.361, *p* < 0.001, *η*_p_^2^ = 0.481), a borderline significant cue × group interaction effect (*F*(1, 50) = 3.811, *p =* 0.057, *η*_p_^2^ = 0.071) and a significant between-group main effect (*F*(1, 50) = 6.340, *p =* 0.015, *η*_p_^2^ = 0.113 for MS 3 duration; and a significant time main effect (*F*(1, 50) = 70.214, *p* < 0.001, *η*_p_^2^ = 0.584), a significant time × cue × group interaction effect (*F*(1, 50) = 13.525, *p =* 0.001, *η*_p_^2^ = 0.213) and a significant between-group main effect (*F*(1, 50) = 7.237, *p =* 0.010, *η*_p_^2^ = 0.126) for MS 3 coverage; there was a significant time × group interaction effect (*F*(1, 50) = 4.566, *p =* 0.038, *η*_p_^2^ = 0.084), a significant cue × group interaction effect (*F*(1, 50) = 7.853, *p =* 0.007, *η*_p_^2^ = 0.136) and a marginal significant time × cue × group interaction effect (*F*(1, 50) = 3.884, *p =* 0.054, *η*_p_^2^ = 0.072) for MS 4 coverage, a significant cue × group interaction effect (*F*(1, 50) = 9.646, *p =* 0.003, *η*_p_^2^ = 0.162) and a significant time × cue × group interaction effect (*F*(1, 50) = 15.791, *p* < 0.001, *η*_p_^2^ = 0.240) for MS 4 occurrence. There was a significant time main effect (*F*(1, 50) = 13.077, *p* = 0.001, *η*_p_^2^ = 0.207) and a significant time × cue × group interaction effect (*F*(1, 50) = 5.439, *p =* 0.024, *η*_p_^2^ = 0.098) for MS 6 duration; a significant time main effect (*F*(1, 50) = 56.794, *p* < 0.001, *η*_p_^2^ = 0.532) and a significant time × cue × group interaction effect (*F*(1, 50) = 13.050, *p =* 0.001, *η*_p_^2^ = 0.207) and a significant between-group main effect (*F*(1, 50) = 7.631, *p =* 0.008, *η*_p_^2^ = 0.132) for MS 6 coverage; a significant time main effect (*F*(1, 50) = 41.832, *p* < 0.001, *η*_p_^2^ = 0.456) and a time × cue × group interaction effect (*F*(1, 50) = 4.721, *p =* 0.035, *η*_p_^2^ = 0.086) and a significant between-group main effect (*F*(1, 50) = 10.456, *p =* 0.002, *η*_p_^2^ = 0.173) for MS 6 occurrence.

**Table 4:** Changes in microstate parameters before and after treatment in the MDD + NSSI group after receiving different treatment.

Microstate parameters		Time main effect *F*(*p*)	Time × Group interaction effect *F*(*p*)	Cue main effect *F*(*p*)	Cue × Group interaction effect *F*(*p*)	Time × Cue interaction effect *F*(*p*)	Time × Cue × Group interaction effect *F*(*p*)	Between-group main effect *F*(*p*)
GEV		0.048 (0.827)	0.011 (0.916)	1.121 (0.295)	0.127 (0.723)	0.599 (0.443)	1.620 (0.209)	1.820 (0.183)
Duration	MS1	1.171 (0.284)	2.438 (0.125)	15.483 (**<0.001**)	4.334 (**0.042**)	3.903 (**0.054**)	2.962 (0.091)	0.052 (0.820)
	MS2	1.408 (0.241)	1.791 (0.187)	6.553 (**0.014**)	6.883 (**0.012**)	15.515 (**<0.001**)	4.778 (**0.034**)	0.354 (0.554)
	MS3	46.361 (**<0.001**)	0.028 (0.869)	0.718 (0.401)	3.811 (**0.057**)	4.362 (**0.042**)	3.576 (0.064)	6.340 (**0.015**)
	MS6	13.077 (**0.001**)	1.571 (0.216)	0.049 (0.826)	0.739 (0.394)	2.092 (0.154)	5.439 (**0.024**)	1.201 (0.278)
Coverage	MS1	0.002 (0.962)	0.426 (0.517)	14.114 (**<0.001**)	2.628 (0.111)	1.814 (0.184)	0.717 (0.401)	0.058 (0.810)
	MS2	1.493 (0.227)	3.298 (0.075)	2.314 (0.134)	12.737 (**0.001**)	10.257 (**0.002**)	2.568 (0.115)	0.022 (0.883)
	MS3	70.214 (**<0.001**)	0.163 (0.688)	5.127 (**0.028**)	1.176 (0.283)	14.069 (**<0.001**)	13.525 (**0.001**)	7.237 (**0.010**)
	MS4	2.133 (0.150)	4.566 (**0.038**)	6.237 (**0.016**)	7.853 (**0.007**)	8.784 (**0.005**)	3.884 (**0.054**)	0.409 (0.525)
	MS6	56.794 (**<0.001**)	0.882 (0.352)	2.177 (0.146)	0.990 (0.325)	6.529 (**0.014**)	13.050 (**0.001**)	7.631 (**0.008**)
Occurrence	MS4	0.931 (0.339)	3.394 (0.071)	2.984 (0.090)	9.646 (**0.003**)	13.098 (**0.001**)	15.791 (**<0.001**)	0.038 (0.846)
	MS6	41.832 (**<0.001**)	0.272 (0.604)	9.005 (**0.004**)	2.359 (0.131)	2.604 (0.113)	4.721 (**0.035**)	10.456 (**0.002**)

Further simple effects analysis revealed that MS 1 duration under negative emotional cues was significantly different before and after the medication combined with rTMS treatment (104.191 ± 54.232 vs. 47.635 ± 35.020, *p* = 0.002), and MS 1 duration under negative emotional cues in medication combined with rTMS treatment group (47.635 ± 35.020 vs. 97.774 ± 82.159, *p* = 0.011) was significantly different compared to the medication treatment group. MS 2 duration and coverage under negative emotional cues were significantly different before and after medication combined with rTMS treatment (77.318 ± 40.961 vs. 154.095 ± 82.002, *p* = 0.001; 0.095 ± 0.072 vs. 0.166 ± 0.077, *p* = 0.003), MS 2 duration and coverage under negative emotional cues in medication combined with rTMS treatment group (154.095 ± 82.002 vs. 107.484 ± 86.773, *p* = 0.011; 0.166 ± 0.077 vs. 0.105 ± 0.086, *p* = 0.012) were significantly different compared to the medication treatment group. MS 4 coverage and occurrence under negative emotional cues were significantly different before and after medication combined with rTMS treatment (0.106 ± 0.068 vs. 0.041 ± 0.444, *p* = 0.001; 1.349 ± 0.557 vs. 0.516 ± 0.491, *p* < 0.001), and MS 4 coverage and occurrence under negative emotional cues in medication combined with rTMS treatment group (0.041 ± 0.444 vs. 0.100 ± 0.073, *p* = 0.002; 0.516 ± 0.491 vs. 1.210 ± 0.675, *p* < 0.001) were significantly different compared to the medication treatment group. MS 3 duration and coverage under negative emotional cues were significantly different before and after medication treatment (232.608 ± 64.021 vs. 123.589 ± 74.201, *p* < 0.001; 0.466 ± 0.062 vs. 0.233 ± 0.132, *p* < 0.001), and significantly different before and after medication combined with rTMS treatment (232.608 ± 64.021 vs. 123.589 ± 74.201, *p* < 0.001; 0.449 ± 0.125 vs. 0.321 ± 0.101, *p* < 0.001). MS 3 duration and coverage under negative emotional cues in medication combined with rTMS treatment group (150.124 ± 61.809 vs. 123.589 ± 74.201, *p* < 0.001; 0.321 ± 0.101 vs. 0.232 ± 0.132, *p* = 0.012) were significantly different compared to the medication treatment group. MS 6 duration, coverage, and occurrence under negative emotional cues were significantly different before and after medication treatment (75.172 ± 41.462 vs. 134.685 ± 78.014, *p* = 0.001; 0.083 ± 0.055 vs. 0.270 ± 0.125, *p* < 0.001; 0.995 ± 0.545 vs. 2.124 ± 0.982, *p* < 0.001) and significantly different before and after medication combined rTMS treatment (80.976 ± 80.843 vs. 126.873 ± 80.213, *p* = 0.032; 0.085 ± 0.085 vs. 0.207 ± 0.114, *p* < 0.001; 0.873 ± 0.721 vs. 1.667 ± 0.697, *p* < 0.001) were significantly different.

### Correlation analysis

We performed a correlation analysis of the differences in scale scores and microstate parameters before and after treatment between the medication combined with rTMS treatment group and the medication treatment group and did not find a correlation.

## Discussion

In this study, we first investigated the performance of depression, NSSI, and microstate parameters in MDD adolescents with NSSI, MDD adolescents, and healthy adolescents exposed to neutral emotional cues and negative emotional cues. We found that MDD adolescents with NSSI had significant depressive symptoms and NSSI symptoms. In terms of microstates, we found that MDD adolescents with NSSI under negative emotional cues had significantly greater duration and coverage of MS 3 than those of healthy adolescents, significantly greater coverage and occurrence of MS 4 than those of MDD adolescents and healthy adolescents, and significantly smaller duration, coverage, and occurrence of MS 6 than those of MDD adolescents and healthy adolescents. Moreover, compared with neutral emotional cues, MDD adolescents with NSSI showed significantly greater duration and coverage of MS 3 and significantly less occurrence of MS 6 with negative emotional cues. This suggests that negative emotional stimuli induce more pronounced changes in microstate parameters in MDD adolescents with NSSI compared to neutral emotional stimuli than adolescents in the other two groups.

We then treated MDD adolescents with NSSI in two different methods and found that both two treatment methods significantly improved depression scores and NSSI scores, with medication combined with rTMS showing lower depression scores and NSSI scores compared to medication. In terms of microstates, we found that both two treatment methods showed significant pre- and post-treatment changes in duration and coverage of MS 3, and duration, coverage, and occurrence of MS 6 under negative emotional cues, with a significant decrease in MS 3 indicators and a significant increase in MS 6 indicators after treatment compared to pre-treatment. Furthermore, there was also a significant change in MS 3 indicators in the medication combined with rTMS treatment group compared to the medication treatment. This suggests that both treatment methods were effective in influencing MS 3 and MS 6 in MDD adolescents with NSSI. In addition to this, the medication combined with rTMS treatment group also showed a significant decrease in duration of MS 1, a significant increase in duration and coverage of MS 2, and a significant decrease in coverage and occurrence of MS 4 before and after treatment. Compared to medication treatment, medication combined with rTMS treatment showed a significant decrease in MS 1 duration, a significant increase in MS 2 duration and coverage, and a significant decrease in MS 4 coverage and occurrence. This suggests that medication combined with rTMS treatment can lead to unique alterations in microstate parameters compared to medication alone, which may be the specific neurophysiological mechanism by which rTMS affects MDD adolescents with NSSI.

Microstate analysis is an important tool for studying the functional brain activity of depressed adolescents with NSSI. Compared with traditional ERP analysis, microstate analysis can present quasi-stable periods of scalp topography over a short window (60–120 ms), reflecting global functional brain activity (37). However, there is no consensus on the classification of microstates. Notably, EEG microstates have been studied in depth both during task performance and in the resting state (i.e., in the absence of a task) (38). Unlike resting-state EEG microstates, task-state EEG microstates have more than four classifications, which are associated with the task, and the polarity of the topographic map and the time course of the EEG (23, 24). In this study, the ERP time course was partitioned into several time-stable topographies, identified as microstates specific to different time processes of the brain during the execution of the task, and corroborated with the average ERP waveforms of the midline electrodes in terms of time course. MS 3 and MS 6 occur mostly from 200 ms before stimulation to 100 ms after stimulation and after 700 ms after stimulation, reflecting a transition from resting brain activity to specific task activity, or a gradual convergence from an active state to a resting state. This is an important stage of functional transition in the brain, as shown in the topographic map showing activation of prefrontal regions of the brain. In the cross-sectional analysis of this study, we found that MDD adolescents with NSSI had altered MS 3 and MS 6 parameters compared to MDD adolescents and healthy adolescents, and that both medication and medication combined rTMS treatments were able to modulate impaired MS 3 and MS 6 indicators, but MS 3 and MS 6 were not specific in the comparison of the two treatment methods, so MS 3 and MS 6 were considered to be indicators with generalized neurophysiological indicators. MS 4 appeared mainly between 100 ms and 300 ms after stimulation, and the topography showed activation in the occipitotemporal region of the brain. The ERP component that appears during this time period is the N250 component, which reflects the early attention and monitoring process of the brain to the received stimulation. In our previous study (39), we found that MDD adolescents with NSSI exhibited an increase in N250 amplitude (absolute value), indicating the presence of impaired early attention and monitoring function in MDD adolescents with NSSI. In this study, we found an increase in MS 4 coverage and occurrence in MDD adolescents with NSSI compared to the other two groups, reconfirming this view from a microstate perspective. In the time period from 300 ms to 500 ms after stimulation, the microstates were mainly reflected in MS 1 and MS 2, which were shown on the topographic map as activation in the parieto-occipital region of the brain, and the topographic map of MS 2 showed more pronounced activation. The ERP component during this time period is the P300 component, which reflects the brain’s cognitive executive processes in response to stimuli. Our previous study (39) found that MDD adolescents with NSSI showed an increase in P300 amplitude, but in the present study, we did not find significant changes in MS 1 and MS 2 parameters in the cross-sectional analysis. For this result, we tried to explain that although the main ERP component present during this time period is P300, there may be other ERPs, and therefore, MS 1 and MS 2 cannot be directly equated to P300 and need to be considered synergistically.

Repetitive transcranial magnetic stimulation was approved by the FDA in 2008 for the treatment of major depression and has been well-documented in numerous studies as a promising treatment for adolescents with MDD (17). However, there is a paucity of research using rTMS intervention with adolescents with NSSI, and its neuromodulatory mechanisms are still not clearly elucidated. A recent study on rTMS-targeted therapy showed that high-frequency stimulation of the left dorsolateral prefrontal region can reduce depressive symptoms (40). Therefore, in the present study, we chose the left dorsolateral prefrontal region as the target site for the rTMS intervention. We conducted two different treatment regimens for MDD adolescents with NSSI, medication alone and medication combined with rTMS. We found that the improvement in clinical symptoms differed between treatments, with patients receiving medication combined with rTMS showing more significant improvement in depressive symptoms and a significant reduction in the occurrence of NSSI. In addition, the changes in microstate parameters exhibited by MDD adolescents with NSSI differed between treatments, with the changes in MS 3 and MS 6 common to both treatment methods, and medication combined with rTMS treatment also had an effect on MS 1, MS 2, and MS 4. We found that medication combined with rTMS treatment downregulated MS 4 parameters more significantly than medication treatment, and that medication combined with rTMS treatment significantly decreased MS 1 parameters and significantly increased MS 2 parameters compared with medication treatment. MS 1 and MS 2 were unchanged in the cross-sectional analysis, but in the longitudinal analysis, we found that medication combined with rTMS treatment was able to harmonize the parameters of MS 1 and MS 2 for better effects in addition to down-regulating the originally increased MS 4 parameters, which may be a potential neural mechanism for the more pronounced effect of medication combined with rTMS treatment than medication treatment. In a recent study (41) of rTMS in schizophrenia microstates, changes in EEG microstate parameters following rTMS were associated with improved symptoms in schizophrenia patients, suggesting that changes in EEG microstates may be a potentially effective indicator of symptom improvement with rTMS. Therefore, we suggest that changes in microstate indicators can be a key neuromodulatory target for the improvement effect of rTMS.

In this study, we compared changes in microstate parameters in MDD adolescents with NSSI, MDD adolescents, and healthy adolescents when exposed to neutral and negative emotional cues, and found differences in microstate parameters that provide a new neurophysiological perspective on the occurrence of NSSI behaviors in MDD adolescents. We also evaluated the improvement effects and changes in microstate parameters in MDD adolescents with NSSI with two different treatments, in which medication combined with rTMS treatment showed more significant improvement in depressive symptoms and NSSI symptoms compared to medication treatment, providing reliable evidence for optimization of treatment in MDD adolescents with NSSI and neurophysiological evidence for the neuromodulatory effect of rTMS treatment.

Although we attempted to optimize the study design as much as possible, there are several limitations that should be noted: First, although NSSI behaviors not only occur in depressed patients, given the impact of patient population heterogeneity on the study, we only chose MDD was as the primary diagnosis and therefore, our results are not applicable to those who do not meet the primary diagnosis of MDD despite having NSSI behaviors. Second, the analysis of baseline demographic data in this study found that the age and sex of subjects in the MDD + NSSI group did not match the other two groups, mainly due to the fact that NSSI behaviors were more common among younger adolescents and were more prevalent in females, which is consistent with the epidemiological characteristics of NSSI. In the cross-sectional microstate analysis of this study, we included age and sex as covariates in the ANOVA to exclude the effect caused by age and sex mismatch, but a study with a large sample matched for age and sex is still needed in the follow-up study has confirmed the results. Third, due to the COVID-19 epidemic, we were somewhat affected in collecting subjects, and some patients were unable to undergo post-intervention EEG acquisition for reasons such as traffic control under the epidemic, so the sample size of MDD adolescents with NSSI receiving both two treatments was insufficient, and future longitudinal studies with larger sample sizes are needed. Fourth, we conducted a correlation analysis between the difference in clinical symptoms and the difference in microstate parameters before and after treatment for MDD adolescents with NSSI receiving different treatment methods, and found no correlation, suggesting that although we found significant changes in microstate characteristics, there was no significant correlation with clinical scales, which may be due to the small sample size of the longitudinal analysis, and correlations may exist when the sample size is increased in the future. Fifth, there are still some shortcomings in the choice of the intervention area for rTMS, our current choice of intervention area is the left dorsolateral prefrontal region, but it may ignore the variability caused by head size, the best option is to use functional MRI to guide the implementation of this area, but the high cost of functional MRI does not allow it to be widely used in clinical practice. For this reason, we used the EEG International 10/20 system to try to avoid the effects of head size differences.

## Conclusion

The results of our study indicate that MDD adolescents with NSSI show abnormal MS 3, MS 4, and MS 6 parameters when exposed to negative emotional stimuli compared to MDD adolescents and healthy adolescents, suggesting a potential neurophysiological mechanism for the occurrence of NSSI behavior in MDD adolescents with NSSI. We further explored the optimization of treatment for MDD adolescents with NSSI, and found the effects of rTMS treatment on MS 1, MS 2, and MS 4 parameters in MDD adolescents with NSSI, providing microstate evidence for the moderating effect of rTMS.

## Data availability statement

The raw data supporting the conclusions of this article will be made available by the authors, without undue reservation.

## Ethics statement

The studies involving human participants were reviewed and approved by Ethics Committee of the First Affiliated Hospital of Chongqing Medical University. Written informed consent to participate in this study was provided by the participants’ legal guardian/next of kin. Written informed consent was obtained from the minor(s)’ legal guardian/next of kin for the publication of any potentially identifiable images or data included in this article.

## Author contributions

LZ conceived the structure of the manuscript and wrote the manuscript. LZ, XH, XP, LM, JH, RC, and ZJ did EEG. CZ, JL, JX, QZe, LD, QZh, and SH prepared the samples. LZ and DZ wrote the code, analyzed the data, and discussed to solve the problem. WW and LK critically reviewed the manuscript. All authors contributed to the article and approved the submitted version.

## Funding

Funding for this study was provided by a grant (81971286) from the National Natural Science Foundation of China (NSFC). And this study was also supported by a grant (cstc2020jcyj-msxmX0222) from the Natural Science Foundation of Chongqing and a grant (CSTC2021jscx-gksb-N0002) from the Science and Technology Bureau of Chongqing. This work was supported by a grant (2023QNXM050) from Chongqing medical scientific research youth project (Joint project of Chongqing Health Commission and Science and Technology Bureau).

## Conflict of interest

The authors declare that the research was conducted in the absence of any commercial or financial relationships that could be construed as a potential conflict of interest.

## Publisher’s note

All claims expressed in this article are solely those of the authors and do not necessarily represent those of their affiliated organizations, or those of the publisher, the editors and the reviewers. Any product that may be evaluated in this article, or claim that may be made by its manufacturer, is not guaranteed or endorsed by the publisher.
